# Triple-Vessel Percutaneous Coronary Revascularization In Situs Inversus Dextrocardia

**DOI:** 10.4061/2010/606327

**Published:** 2010-06-10

**Authors:** Nikolaos Kakouros, Sundip J. Patel, Simon Redwood, Balvinder S. Wasan

**Affiliations:** ^1^Johns Hopkins University, Baltimore, MD 21205, USA; ^2^Department of Cardiology, Queen Elizabeth Hospital, Stadium Road, Woolwich, London, England, SE18 4QH, UK; ^3^Department of Cardiology, St. Thomas' Hospital, Lambeth Palace Road, London SE1 7EH, UK

## Abstract

Dextrocardia with situs inversus occurs in approximately one in 10,000 individuals of whom 20% have primary ciliary dyskinesia inherited as an autosomal recessive trait. These patients have a high incidence of congenital cardiac disease but their risk of coronary artery disease is similar to that of the general population. We report what is, to our knowledge, the first case of total triple-vessel coronary revascularization by percutaneous stent implantation in a 79-year-old woman with situs inversus dextrocardia. We describe the successful use of standard diagnostic and interventional guide catheters with counter rotation and transversely inversed image acquisition techniques. The case also highlights that the right precordial pain may represent cardiac ischemia in this population.

## 1. Case Report

**Figure 1:**
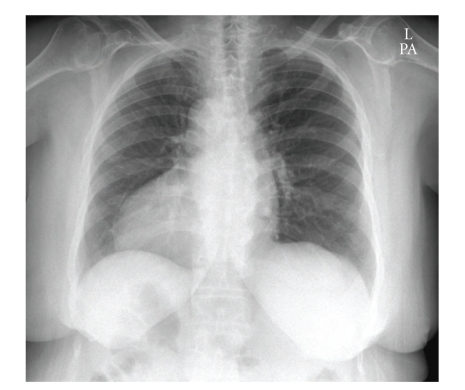
Chest radiograph confirming dextrocardia with right-sided aortic arch and right-sided gastric bubble.

A 79-year-old Caucasian woman presented with a two-week history of worsening right precordial chest pain on exertion. Risk factors for ischemic heart disease included hypercholesterolemia, hypertension, and high body-mass index. Past medical history was notable for pulmonary embolus 11 years previously and hiatus hernia. Clinical examination revealed a right-sided apex impulse, and chest radiograph confirmed dextrocardia with right-sided aortic arch (Figure 1). ECGs showed anterolateral ST-segment depression during pain. Initial management was with aspirin, clopidogrel, low-molecular-weight heparin, and beta-blocker. Serial Troponin T measurement was within normal limits. A myocardial perfusion scan showed reduced uptake at the anteroseptum and apex that appeared nonreversible. The patient, however, continued to have chest pain on minimal exertion and was offered coronary angiography.

**Figure 2:**
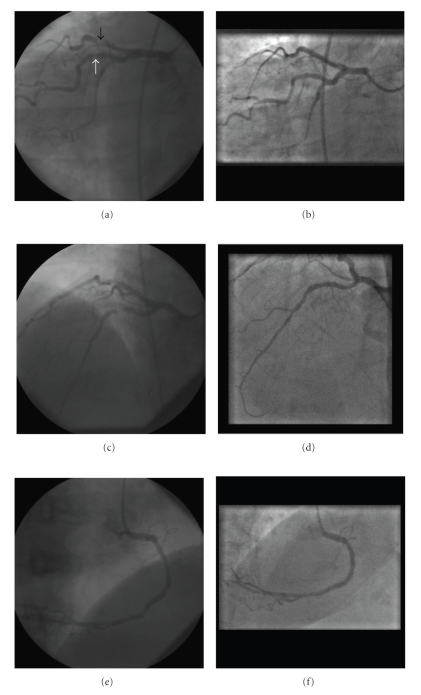
Coronary angiograms pre and post coronary intervention (left and right columns, resp.). Panels (a, b): Left anterior Oblique (LAO) caudal view showing lesion in large obtuse marginal (white arrow) and severe stenosis of LAD (black arrow), Panels (c, d): PA cranial view showing severe LAD stenosis prior to (c) and post intervention (d). Panels (e, f): Left-sided right coronary. artery in Right anterior oblique (RAO) view.

Coronary angiography was performed via the right femoral artery. The anatomical left coronary system (right sided) was cannulated with a 6-French Judkins Left 4 catheter. The transverse angles (right/left obliques) were reversed whilst keeping the cranial/caudal tilts the same. A critical lesion was noted in the mid-left anterior descending (LAD) artery with a further significant stenosis at the proximal part of a large first obtuse marginal branch (Figure 2). The left-sided anatomical right coronary artery (RCA) was cannulated with a 6-French Judkins Right 4 catheter using counterclockwise rotation. There was a long segment of significant disease in the mid vessel and a focal lesion just before the crux (Figure 2). 

Revascularization options were subsequently discussed with the patient who was not keen on surgical intervention and opted for percutaneous revascularization. A 6 F EBU (Extra Backup) 3.5 guide catheter (Launcher; Medtronic Corp., Minneapolis) was used to engage the left main stem. The PA Cranial radiographic view was chosen for the LAD—the vessel appearing as a “mirror image” from its usual orientation. The lesion was crossed with a BMW wire (Guidant Corp., California) and, following predilatation with a 2 mm × 12 mm Maverick balloon (Boston Scientific Corp., Massachusetts), a 2.75 mm × 20 mm Taxus Liberté (Boston Scientific Corp.) stent was deployed. Using the same guide catheter and wire in the LAO Caudal view (Figure 2), the obtuse marginal lesion was crossed and a 3.0 mm × 8 mm bare metal Liberté stent (Boston Scientific Corp.) was deployed. A 6F right Judkins guide catheter was used to engage the right coronary artery. Using the straight RAO view, a 2.75 mm × 12 mm Liberté stent was placed in the mid-to-distal RCA and a 3.0 mm × 24 mm Liberté stent in the proximal-to-mid RCA. An excellent angiographic result was obtained at all sites (Figure 2—right column). Dual antiplatelet therapy with aspirin and clopidogrel was recommended for one year. The patient was reviewed 3 months later and reported that her quality of life had greatly improved as she no longer experienced chest pain. A repeat diagnostic angiogram at 6 months revealed widely patent stents in all three vessels.

## 2. Discussion

Individuals with situs inversus dextrocardia have a high incidence of congenital heart disease [1] but their risk of coronary artery disease is the same as that in the general population [2]. This case documents what is, to our knowledge, the first triple-vessel coronary revascularization by stent implantation in a patient with situs inversus dextrocardia. 

Presentation of patients with dextrocardia with variant myocardial ischemia symptoms of right-sided chest pain has been previously noted [3–5]. The reason for this atypical presentation is unclear. Situs inversus has been previously shown to be associated with abnormal neural axis development [6] and this may lead to variant visceral pain perception. Afferent fibers from the heart travelling along the sympathetic trunks of the neck and thorax may enter the higher thoracic levels (T1 to T4/5) from the right rather than the left side, thus associating with spinal ganglia and spinal cord segments receiving sensory impulses from the right side of the body. This may also explain previous reports of ischemic pain referral down the right shoulder and arm in these patients [7–9]. Pain localization from abdominal viscera is also discrepant and suggests that peripheral nerve route transposition occurs in about 50% of cases of situs inversus [10]. Consequently, patients with dextrocardia may present with pain referred to either the left or the right side of the body.

In the first reported case of percutaneous transluminal coronary angioplasty in dextrocardia with situs inversus, Moreyra et al. found Judkins catheters unhelpful [11]. It has since been suggested that the suitability of diagnostic and interventional guide catheters can be predicted by the aortic arch position [12–14]. In our experience, however, even in the presence of right-sided aortic arch, the left Judkins catheter can be used to cannulate the right-sided morphologically left coronary artery. Similarly, the right Judkins catheter manipulated to the mirror of its usual position can cannulate the left-sided morphological RCA. Notably, catheter rotation (torquing) is in the opposite direction of that employed for normal cardiac anatomy. Counterclockwise rotation is, for example, required to engage the left-sided RCA with the right Judkins catheter. Furthermore, we demonstrate that engagement of the coronary arteries with other standard catheters (such as EBU) is possible in this setting. Successful employment of Judkins catheters has been previously reported in a case of dextroversion (dextrocardia with normally related atria and left-sided aortic arch). This is not surprising as in these patients the coronary artery ostia lie close to their usual positions [15]. Consequently, contrary to previous reports, we suggest that the standard preformed catheters may be employed in cases of dextrocardia with both right- and left-sided aortic arch position. 

**Figure 3:**
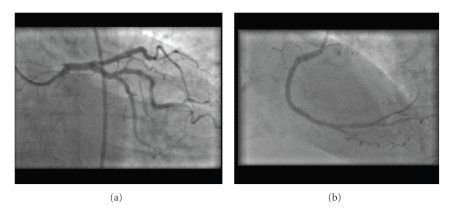
Double-inversion technique. Panel (a): LAO caudal view (as in Figure 2(a)) with additional horizontal inversion demonstrating apparent “normal” RAO caudal angiographic appearances. Note, also, engagement of the EBU catheter. Panel (b): RAO view of the left-sided right coronary artery with additional horizontal inversion mimicking a conventional LAO view.

A double-inversion technique of reversed image acquisition and horizontal inversion of on-screen display has been previously described [16]. In our case, angiography and intervention was performed without on-screen reversal of the images. It is still necessary, however, to reverse the transverse angulations whilst maintaining the common cranial/caudal sagittal tilts. A right anterior oblique caudal projection, for example, produces a mirror image “spider view”. Although horizontal image inversion in conjunction with horizontally reversed image acquisition (double inversion) makes the coronary tree appear “normal” (see Figure 3), it also artificially reverses the on-screen response of catheters and angioplasty wires to normal manipulation (torquing). Consequently, although it may aid image interpretation, we found this technique unhelpful for percutaneous intervention. 

In summary, we report the first case of triple-vessel coronary revascularization by percutaneous stent implantation in situs inversus dextrocardia. In this case of a right-sided aortic arch, Judkins and EBU catheters, transversely inversed image acquisition, and counter rotation of catheters were successfully used with no complications. Finally, we remind the reader that right precordial pain may represent cardiac ischemia in this population and should not be dismissed as being “atypical”.
